# Mismatched light and temperature cues disrupt locomotion and energetics via thyroid-dependent mechanisms

**DOI:** 10.1093/conphys/coaa051

**Published:** 2020-06-11

**Authors:** Amélie Le Roy, Frank Seebacher

**Affiliations:** School of Life and Environmental Sciences A08, University of Sydney, NSW 2006

**Keywords:** acclimation, climate change, metabolic scope, mitochondria, phenotypic plasticity

## Abstract

Animals integrate information from different environmental cues to maintain performance across environmental gradients. Increasing average temperature and variability induced by climate change can lead to mismatches between seasonal cues. We used mosquitofish (*Gambusia holbrooki*) to test the hypotheses that mismatches between seasonal temperature and light regimes (short days and warm temperature and vice versa) decrease swimming performance, metabolic rates and mitochondrial efficiency and that the responses to light and temperature are mediated by thyroid hormone. We show that day length influenced thermal acclimation of swimming performance through thyroid-dependent mechanisms. Oxygen consumption rates were influenced by acclimation temperature and thyroid hormone. Mitochondrial substrate oxidation rates (state three rates) were modified by the interaction between temperature and day length, and mitochondrial efficiency (P/O ratios) increased with warm acclimation. Using P/O ratios to calibrate metabolic (oxygen consumption) scope showed that oxygen consumption did not predict adenosine triphosphate (ATP) production. Unlike oxygen consumption, ATP production was influenced by day length in a thyroid-dependent manner. Our data indicate that oxygen consumption alone should not be used as a predictor of ATP production. Overall, the effects of thyroid hormone on locomotion and energetics were reversed by mismatches such as warm temperatures on short days. We predict that mid to high latitudes in North America and Asia will be particularly affected by mismatches as a result of high seasonality and predicted warming over the next 50 years.

## Introduction

Combining information from several cues simultaneously is likely to give a more reliable prediction of the upcoming conditions compared to responses to a single cue only (Bradshaw and Holzapfel, 2008). For example, many ectotherms respond to thermal and photoperiodic cues and can reversibly acclimate their phenotype in response to persistent environmental conditions (Day and Butler, 2005; Guderley, 2004; Schulte *et al.*, 2011), and these responses are at least partially driven by endocrine signalling (Falcón *et al.*, 2007; Little *et al.*, 2013; Nakane and Yoshimura, 2019). Thyroid hormone, in particular, is important in temperature acclimation of muscle function and metabolism (Little *et al.*, 2013; Little and Seebacher, 2013) and in regulation of responses to photoperiod (Nakane and Yoshimura, 2019). Additionally, it regulates mitochondrial bioenergetics (Harper and Seifert, 2008), and it can alter mitochondrial leak (State 4) respiration and efficiency via genomic and non-genomic mechanisms (Harper and Brand, 1993; Lanni *et al.*, 2016). Thyroid hormone is therefore a likely candidate that synchronizes responses to temperature and light.

Current environmental change may lead to mismatches between thermal and photoperiodic information. For example, winter and spring temperatures are increasing as a result of climate warming while photoperiod remains unaltered, and light at night causes artificially long days at any temperature regime (Gaston, 2018; Garrett *et al.*, 2019). To predict responses of animals to environmental change requires understanding of how seasonal cues interact to modulate physiological processes and how this interaction is regulated. Day length can interact with temperature to modify acclimation of physiological capacity. For example, a mismatch between temperature and photoperiod during cold acclimation decreased sustained swimming performance in brown trout (*Salmo trutta*) (Day and Butler, 2005). In mosquitofish (*Gambusia holbrooki*), regulation of thermal acclimation of metabolic scope differed between spring-born (i.e. exposed to short days) and summer-born (long days) fish (Seebacher *et al.*, 2014). Metabolic scope is usually calculated as the difference between maximal and maintenance rates of whole body oxygen consumption to estimate energy (adenosine triphosphate, ATP) available for activity (Clark *et al.*, 2013). The relationship between oxygen consumption and energy availability is based on the approximation that the amount of ATP produced per atom of oxygen consumed is constant across individuals and conditions (Brand, 2005). However, mitochondrial bioenergetics change with temperature (Chung and Schulte, 2015; Khan *et al.*, 2014), and potentially with photoperiod. In the red muscle of rainbow trout, for example, the increase in mitochondrial substrate oxidation rate (State 3 respiration rate) typically associated with cold acclimation was enhanced by acclimation to short day length (Martin *et al.*, 2009). Additionally, the number of oxygen atoms used to phosphorylate one ADP to ATP is not constant (Brand, 2005). Calibrating whole body oxygen consumption with mitochondrial efficiency (i.e. the number of oxygen atoms used to produce one ATP) will therefore provide a better estimate of how environmental change affects animal energetics (Salin, Auer, Rey, Selman & Metcalf, 2015).

We conducted a fully factorial experiment (using mosquitofish, *G. holbrooki*) in which we manipulated thyroid hormone status, day length, chronic acclimation temperature and acute test temperature to test the hypotheses that (i) mismatches between day length and temperature compromise ATP production and locomotor performance; in particular, (ii) short day length enhances the effect of cold acclimation, and the capacity for cold acclimation is reduced with long days; (iii) induced hypothyroidism reduces compensatory responses to cold temperature and to light cues, but it decreases leak respiration in mitochondria (Harper and Seifert, 2008) and thereby increases ATP production capacity. We measured sustained locomotor performance, oxygen consumption and the bioenergetics of muscle mitochondria. Oxygen consumption and mitochondrial bioenergetics were measured in the same individuals, which allowed us to calculate metabolic scope in terms of ATP produced as well as of oxygen consumed.

## Materials and methods

### Animals and treatments

All experiments were carried out with the approval of the University of Sydney Animal Ethics Committee (approval number: 2018/1139). Newborn *G. holbrooki* were collected in late spring (November) from Manly Dam, Australia (33°78 S; 151°26 E) using a large dip net. Fish were allowed to grow for 2 months at 23°C and under a 14:15-h light/9:45-h dark light cycle (14:15L/9:45D, intermediate conditions). Fish (*n* = 200) were then divided into two different light treatments, short days (SD, 12:30L/11:30D) and long days (LD, 16L/8D). Within each light treatment, fish were separated into a cold (18°C) and a warm acclimation treatment (28°C). See below for sample sizes used in each treatment. The SD/18°C treatment represents late winter/early spring conditions (late August), and the LD/28°C treatment represents summer conditions (late December) at the collection site. Within each of the four treatments, fish were further separated between a normothyroid (control) group and a hypothyroid treatment group. We induced hypothyroidism following a published protocol used successfully on zebrafish (*Danio rerio*) (Little *et al.*, 2013). Briefly, we maintained the water at 0.3 mM of propylthiouracil (Sapphire Bioscience, Australia) dissolved in DMSO (0.05% final concentration), which blocked the production of T4 by the thyroid gland, and 5 μM of iopanoic acid (Thermo Fisher Scientific Inc., Australia) dissolved in ethanol (0.025% final concentration), which prevented the conversion of the inactive T4 to the active T3 and T2 by inhibiting deiodinase enzymes. The normothyroid groups were maintained at 0.05% DMSO and 0.025% ethanol. Within each treatment, fish were dispersed across four experimental tanks. Fish stayed in their eight respective treatments for 4 weeks, before we measured swimming performance, oxygen consumption rates (resting and active) and mitochondrial bioenergetics at two acute test temperatures (18 and 28°C). We used different individuals to measure swimming performance, but we measured resting and maximal rates of oxygen consumption and mitochondrial bioenergetics in the same individuals. We measured each individual repeatedly at the two acute test temperatures; the order of acute test temperatures was alternated, and fish had at least 24-h rest in their home tank between measurements (except for resting oxygen consumption measurements).

We verified the efficacy of the hypothyroid treatment by testing whether supplementation of hypothyroid fish with the active forms of thyroid hormone, 3,5-diiodothyronine (T2), and 3,5,3′-triiodothyronine (T3), counteracts the effect of induced hypothyroidism (Little *et al.*, 2013). We therefore induced hypothyroidism in an additional group of SD/18°C fish (*n* = 11), which we supplemented daily with 10 nM of T2 and T3 (Sigma–Aldrich, Castle Hill, Australia) for 4 weeks after which we measured swimming performance. We compared these fish to the SD/18°C hypothyroid and control fish of the main experiment by permutational analyses (see below) and confirmed the efficacy of our hypothyroid treatment (Supporting Data; Fig. S1).

### Swimming performance

We measured maximal swimming capacity as critical sustained swimming speed (*U*_crit_) according to published protocols (Hammill *et al.*, 2004). Fish (*n* = 13–16 per treatment group) were placed in a cylindrical clear Perspex tube (150-mm length, 32-mm diameter) tightly fitted over the intake end of a submersible inline pump (12 V DC, iL500, Rule, Hertfordshire, UK) and submerged in a plastic tank (645 × 423 × 276 mm). The flow was adjusted with a variable DC power source (NP9615; Manson Engineering Industrial, Hong Kong) connected to the pump and was measured in real time using a flow meter (DigiFlow 6710 M, Savant Electronics, Taichung, Taiwan). A bundle of hollow straws fitted at the inlet helped maintain a laminar flow through the flume. Fish were transferred to the flume within the tank kept at the appropriate acute test temperature and swam at an initial flow rate of 0.06 m s^−1^ for 20 min. Flow was then increased by 0.02 m s^−1^ every 5 min. When fish first fell back on the grid separating the flume from the pump, we turned the flow off for 10 s and then resumed the previous flow rate; when fish fell back on the grid a second time, they were defined to be exhausted, and we stopped the trial and recorded the time spent at the final speed to calculate *U*_crit_ (Hammill *et al.*, 2004). Fish standard length was measured after completion of the swimming trials, and *U*_crit_ was expressed in body length per second (BL s^−1^). Note that we avoided any order effects by alternating treatments in consecutive swimming trials.

### Oxygen consumption

Resting oxygen consumption (MO_2rest_) rates were measured according to published protocols (Le Roy *et al.*, 2017). Fish (*n* = 16–21 per treatment group) were placed in Perspex cylindrical respirometers (15 mm diameter and 100 mm length, 27 ml volume) immersed in a temperature-controlled water bath and connected to a peristaltic pump (i150, iPumps, Tewkesbury, UK), which circulated water through the chambers. Oxygen concentration inside the chambers was measured using sensor spots (Loligo Systems, Viborg, Denmark) stuck to the inside, halfway along the length of the chambers and monitored by fibre optic cables linked to an oxygen meter (Witrox, Loligo Systems, Viborg, Denmark). We monitored 15 fish in individual chambers concurrently. Fish were allowed to rest undisturbed for 2 h before trials, which is sufficient time to overcome the effects of handling stress (Seebacher *et al.*, 2016). To measure oxygen consumption, pumps were turned off, and the decrease in oxygen concentration inside the sealed chamber was monitored for 30 min. After the 30-min recording period, we turned the peristaltic pump on again, and gradually, over 30 min changed the water temperature to the second test temperature. After the fish spent 15 min at the new test temperature, we measured oxygen consumption as described above. During measures of oxygen consumption, fish were monitored with a camera (HD1080P, Logitech, USA) connected to the same computer used for data acquisition to monitor movement. After measurements, fish were weighed on an electronic balance. We used the slopes of the decrease in oxygen concentration to calculate resting oxygen consumption rates in μmol O_2_ g^−1^ min^−1^.

After measuring resting oxygen consumption rates, exercise-induced maximal oxygen consumption rates (MO_2max_) were measured in the same fish in glass respirometers (130 ml) containing a magnetic stir bar that was separated from the fish by a mesh partitioning. The respirometer was immersed in a temperature-controlled water bath, which was placed on top of a magnetic stirrer. We controlled the circular flow in the respirometer by adjusting the stirring speed on the magnetic stirrer, and a central plastic column suspended from the lid inside the respirometer helped reduce turbulence. A sensor spot (Loligo Systems, Viborg, Denmark) glued to the inside of the chamber wall measured oxygen concentration and was monitored by a fibre optic cable, connected to an oxygen meter (FIBOX 3, PreSens, Regensburg, Germany). We increased the flow speed inside the chamber until the fish struggled to hold its position in the water column, which we considered to be near maximal swimming speed. We recorded the decrease in oxygen concentration at this flow speed for ~10 min and observed fish directly to ensure animals were swimming during that period. We calculated oxygen consumption rates as above. As for swimming trials, fish from the same treatment were not swum consecutively to avoid order effects, and fish had 24-h rest between measures of maximal oxygen consumption rates at different test temperatures. We calculated oxygen consumption scope (MO_2scope_) as the difference between maximal and resting rates of oxygen consumption (Clark *et al.*, 2013).

### Mitochondrial bioenergetics

Within a week of measuring oxygen consumption, fish were anaesthetized using iso-eugenol (Aqui-S, New Zealand) and decapitated on ice to dissect rostral and tail skeletal (mixed) muscle immediately for measurements of mitochondrial respiration. Mitochondrial measurements were conducted according to published protocols (Ghanizadeh Kazerouni *et al.*, 2016) at 18 and 28°C acute test temperature consecutively for each sample. All chemicals were purchased from Sigma-Aldrich (Castle Hill, Australia). The tail muscle was dissected and homogenized using a Potter–Elvehjem tissue homogenizer in nine volumes of isolation buffer [KCl 140 mM, HEPES 20 mM, MgCl_2_ 5 mM, EGTA 2 mM, ATP 1 mM, BSA (fatty acid free) 0.5 g l^−1^, pH 7 (DosSantos *et al.*, 2013)]. The homogenate was centrifuged at 1400 g for 5 min, and the supernatant containing the mitochondria was set aside while the pellet was resuspended and centrifuged again. The supernatants were combined and centrifuged at 9000 g for 9 min to settle the mitochondria, and the supernatant was discarded. The pellet was resuspended in assay medium [sucrose 110 mM, KCl 60 mM, EGTA 0.5 mM, MgCL_2_ 3 mM, taurine 20 mM, KH_2_PO_4_ 10 mM, HEPES 20 mM, BSA (fatty acid free) 0.5 g l^−1^, pH 7.1 (DosSantos *et al.*, 2013)] at a ratio of 2 ml g^−1^ of tissue. Mitochondrial oxygen consumption was measured in a respiration chamber (Mitocell MT200; Strathkelvin Instruments, North Lanarkshire, UK) with an oxygen electrode (model 1302; Strathkelvin Instruments) connected to an oxygen meter (model 782; Strathkelvin Instruments). We triggered State 2 respiration by adding final concentrations of 5 mM of malate and 2.5 mM of pyruvate. State 3 respiration was induced by adding 0.5 mM of ADP, and we determined natural State 4 when ADP was used up, indicated by stabilisation of oxygen consumption rate 30–50 min after ADP addition. Finally, the addition of 1 μM of carbonyl cyanide-*p*-trifluoromethoxy phenyl hydrazone (FCCP) estimated the maximal uncoupled rates of oxygen uptake and allowed us to verify the integrity of the mitochondrial membrane. Protein concentration of the mitochondrial extract was determined using a Bradford assay (Sigma-Aldrich, Castle Hill, Australia), with BSA as a standard. We estimated the ratio between ATP produced to oxygen consumed (P:O ratio) as the amount of ADP present in the chamber (0.5 mM) divided by the number of oxygen atoms used to consume it. Oxygen use was calculated as the difference in oxygen levels between the beginning of State 3 and the start of State 4. As above, we avoided order effects by alternating treatments when conducting the assays, so that any potential confounding effects, such as differences between batches of chemicals, for example, would have been spread across all treatments.

### ATP production scope

Metabolic rates are commonly measured as whole-animal oxygen consumption rates. Resting oxygen consumption is typically used as a proxy for the energetic cost of maintaining essential cellular functions such as membrane potentials and protein synthesis when animals are inactive, while active oxygen consumption reflects maximal aerobic capacities (Clark *et al.*, 2013; Hulbert and Else, 2000). The difference between active and resting oxygen consumption rates, or metabolic scope, is widely used to estimate the energy (ATP) available for activity (Auer *et al.*, 2017). However, correcting individual oxygen consumption rates for mitochondrial efficiency renders estimates of energy availability more accurate, because the amount of oxygen used to produce an ATP molecule varies (Salin *et al.*, 2015). We therefore multiplied individual metabolic scope by the corresponding P:O ratio to convert oxygen consumed into an estimate of ATP produced; we refer to this calculated variable as ATP production scope. We acknowledge that our estimate of ATP production scope is based on measurements of skeletal muscle only and that there may be bioenergetic differences between organs. However, skeletal muscle makes up most of the body mass in fish (Johnston *et al.*, 2011) so that our estimates are relevant for the whole organisms.

### Statistical analysis

We conducted a fully factorial analysis with day length (short day or long day), thyroid status (hypothyroid or normothyroid), acclimation temperature (18°C or 28°C) and acute test temperature (18°C or 28°C) as fixed factors; all interactions were included, except for the highest, four-way interaction. The identity of the fish was used as a random factor to account for the repeated measures across different test temperatures. Where informative, we included *post hoc* tests of marginal means to help interpret significant interactions. We conducted permutational analyses on all response variables, using the R package ‘lmPerm’ (Wheeler and Torchiano, 2016), because it is robust for small sample sizes and free from assumptions about underlying distributions (Drummond and Vowler, 2012; Ludbrook and Dudley, 1998). Effect sizes [Cohen’s *d*; (Cohen, 1992)] with 95% confidence intervals were used to present the effects of hypothyroidism on our response variables, because we were more interested in the direction of the effect than in its absolute value. We calculated effect sizes so that a positive effect size also indicated a positive effect of thyroid hormone on response variables. We determined bootstrap 95% confidence intervals (Calmettes *et al.*, 2012) for all effect sizes using the R package ‘boot’. The full set of results for all variables and factors is presented in the Supplementary Material (Supplementary Material Figs S2–S8).

We calculated effect sizes for the specific mismatch of warm temperatures at short day length. Hence, we compared the effect of warm conditions (28°C acclimation and test temperatures) at short day lengths, which simulates a warming scenario, to warm conditions at long day lengths, which estimate natural summer conditions. We estimated the global significance of the mismatch (warm conditions at short day lengths) under future climate warming by extracting current and projected future climate data from WorldClim 1.4 and 2.0 using the ‘raster’ package in R. We reasoned that increasing seasonality would increase the impact of mismatches, particularly in regions that are forecast to undergo future warming. We estimated annual seasonality as the difference between the mean monthly minimum and mean monthly maximum temperatures globally between 1970 and 2000 and multiplied this by the estimated increase in mean temperature by 2070 under emission scenario RCP8.5 (Riahi *et al.*, 2011). We scaled the resulting metric to give an index between 0 and 1, where 0 represents no effect and 1 represents the greatest effect globally. Note that model predictions are indicative only, and we used air temperatures in these predictions, which will show the same patterns of fluctuations as surface water temperatures although fluctuations will be dampened in the latter (Piccolroaz *et al*. 2013).

## Results

### Day length influenced thermal acclimation of swimming performance through thyroid-dependent mechanisms

**Figure 1:**
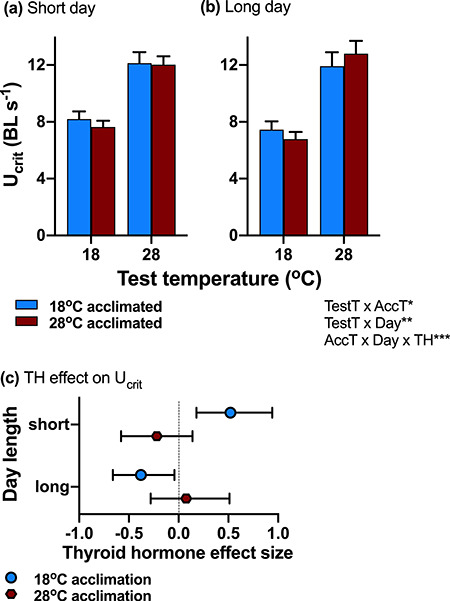
Critical sustained swimming speed. Swimming speed (*U*_crit_) in normothyroid fish was determined by an interaction between acute test temperature and acclimation temperature (red bars = acclimation to 28°C; blue bars = acclimation to 18 C), and an interaction between day length (left panel = short day, right panel = long day) and acute test temperature. (**c**) Effect sizes (Cohen’s *d*) of thyroid hormone on *U*_crit_ (positive effect sizes also indicate a positive effect of thyroid hormone). Thyroid hormone increased *U*_crit_ when cold acclimation temperature (blue symbols = acclimation to 18°C; red symbols = acclimation to 28°C) matched short days, but it decreased *U*_crit_ when there was a mismatch between long days and cold acclimation (interaction between acclimation temperature × day length × thyroid status; marginal means across test temperatures are shown). The significant effects and interactions (thyroid status = TH, day length = day, acclimation temperature = AccT, and test temperature = TestT) are listed on the bottom right (^*^P < 0.05; ^**^P < 0.01: ^***^P < 0.0001). Means ± SE are shown in (**a**) and (**b**), and means ±95% CI in (**c**); sample size was *n* = 13–16 fish per treatment group

At cold test temperatures, cold-acclimated fish had greater sustained swimming performance (*U*_crit_) than warm-acclimated fish (interaction acclimation temperature × test temperatures; Table 1; Fig. 1a and b; Supplementary Material Fig. S2). The interaction between day length and acclimation temperature (Table 1; Fig. 1a and b) indicates that short day exposure enhanced the effects of cold acclimation. However, *post hoc* tests could not resolve differences between treatment groups statistically (all *P* > 0.2). At the cold test temperature, *U*_crit_ was greater on short days than on long days (interaction day length × test temperature; Table 1; Fig. 1a and b; comparison between marginal means *P* = 0.04).

**TABLE 1:** Results from the permutational analysis of whole-animal performance and mitochondrial bioenergetics

Source	*U*_crit_	MO_2rest_	MO_2max_	MO_2s_	S3	S4	P:O	ATP_s_
TestT	**<0.001**	**<0.001**	**<0.001**	**<0.001**	**<0.001**	0.13	0.12	0.089
AccT	0.84	**0.0018**	**<0.001**	**0.017**	0.26	0.36	**<0.001**	**0.028**
Day	0.12	0.94	0.94	0.71	**<0.001**	0.56	0.65	1
TH	1	0.25	0.50	0.44	0.96	0.30	0.46	0.26
TestT:AccT	**0.042**	0.091	1	0.78	0.49	0.80	0.36	0.86
TestT:Day	**0.01**	0.088	0.41	0.50	**<0.001**	0.56	1	1
TestT:TH	0.94	0.98	0.37	0.86	0.69	0.88	0.56	0.73
AccT:Day	**0.03**	0.49	0.96	0.92	**0.02**	0.50	1	1
AccT:TH	0.71	0.63	0.94	0.96	0.40	0.12	0.24	0.94
TH:Day	0.14	0.78	0.39	0.42	1	0.26	0.28	0.53
TestT:AccT:Day	0.84	0.38	0.51	0.46	0.67	0.62	0.76	0.12
AccT:Day:TH	**<0.001**	0.92	0.36	0.078	0.16	1	1	1
Day:TH:TestT	0.73	0.90	0.52	0.55	0.38	0.78	0.068	**0.032**
TestT:AccT:TH	1	**0.039**	0.60	0.29	1	0.82	0.51	0.62

Measured response were critical sustained swimming performance (Ucrit), oxygen consumption during rest (MO2rest) and exercise (MO2max), oxygen consumption scope (MO2s), State 3 (S3) and State 4 (S4) mitochondrial respiration rates, P:O ratios (P:O) and ATP production scope (ATPS). Numerator degrees of freedom = 1 for all factors (TestT: acute test temperature; AccT = acclimation temperature; day = day length; TH = thyroid status) and interactions. Permutational P values are shown and significant effects at P < 0.05 are in bold

The interaction between acclimation temperature and day length was dependent on thyroid hormone (significant three-way interaction; Table 1). Thyroid hormone promoted cold acclimation of swimming performance in fish exposed to short days {positive effect size [Cohen’s *d* (Cohen, 1992)]; Fig. 1c}, but this effect was reversed in fish exposed to long days where thyroid hormone decreased cold acclimation of *U*_crit_ (Fig. 1c).

### Oxygen consumption acclimated to temperature and was influenced by thyroid hormone

**Figure 2:**
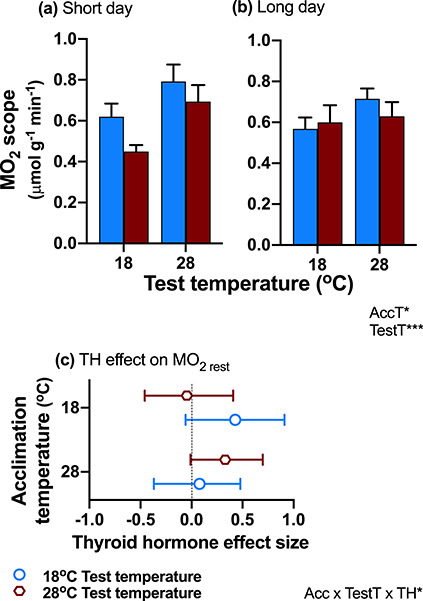
Oxygen consumption. Oxygen consumption scope (MO_2_ scope; **a**, **b**) responded to thermal acclimation (main effect of acclimation; blue bars = acclimation to 18°C; red bars = acclimation to 28°C), and cold-acclimated animals had greater scope, thereby reducing the negative effect of low test temperature. MO_2_ scope also increased with increasing acute test temperature (main effect of test temperature), but there was no significant effect of day length (**c**) Thyroid hormone increased resting metabolic rate when test temperatures matched acclimation temperatures (interaction between test temperature × acclimation temperature × thyroid status; open red circles = 28°C test temperature, open blue circles = 18°C test temperature). Effect sizes (Cohen’s *d*) of thyroid hormone are shown, and positive effect sizes also indicate a positive effect of thyroid hormone. The significant effects and interactions (thyroid status = TH, acclimation temperature = AccT, and test temperature = TestT) are listed on the bottom right (^*^P < 0.05; ^**^P < 0.01: ^***^P < 0.0001). Means ± SE for normothyroid fish are shown in (**a**) and (**b**), and means ±95% CI in (**c**); sample size was *n* = 16–21 fish per treatment group

Resting and exercise-induced maximal rates of oxygen consumption, as well as oxygen consumption scope (maximal-resting rates), increased significantly with increasing acute test temperature and were higher in cold-acclimated compared to warm-acclimated animals (main effects of test temperature and acclimation temperature; Table 1; Fig. 2a and b; Supplementary Material Figs S2–S4). Thyroid hormone increased resting oxygen consumption rates when acclimation and test temperatures matched (three-way interaction; Table 1; Fig. 2c).

### Day length modified thermal responses of mitochondrial substrate oxidation rates

**Figure 3:**
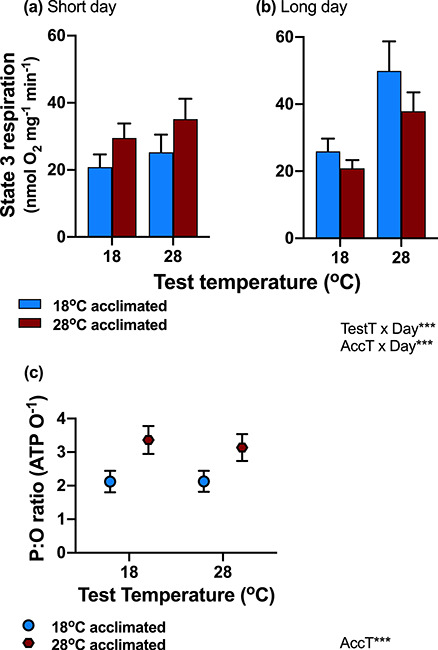
Mitochondrial bioenergetics. A mismatch between acclimation temperature and day length increased State 3 rates (acclimation temperature × day length interaction), which were greater in warm-acclimated animals (red bars) on short days (**a**), and cold-acclimated (blue bars) animals on long days (**b**). The thermal sensitivity of State 3 rates was also greater on long days than on short days (test temperature × day length interaction). The estimated amount of ATP produced for each oxygen consumed (P:O ratio; **c**) was significantly greater (main effect of acclimation temperature) in warm-acclimated (red circles) fish compared to cold-acclimated fish (blue circles). The significant effects and interactions (day length = day, acclimation temperature = AccT and test temperature = TestT) are listed on the bottom right (^*^P < 0.05; ^**^P < 0.01: ^***^P < 0.0001). Means ± SE are shown, and sample size was *n* = 16–21 fish per treatment group

Substrate oxidation (State 3) respiration rates in normothyroid fish were modified significantly by the interaction between day length and acclimation temperature (Table 1; Fig. 3a and b, Supplementary Material Fig. S5). On short days, State 3 rates were higher in fish acclimated to warm temperatures than to cold temperatures, but the reverse was the case on long days (Fig. 3a and b). There was also an interaction between day length and test temperature (Table 1), and State 3 rates were more sensitive to an increase in acute test temperature on long days compared to short days. There was no effect of hypothyroidism on State 3 rates (Table 1).

Leak (State 4) respiration rates were not affected significantly by any of the experimental factors (Table 1; Supplementary Material Fig. S6).

### Warm acclimation increased mitochondrial efficiency (P:O ratios)

Warm-acclimated fish had greater P:O ratios (i.e. greater mitochondrial efficiency indicated by the lower estimated amount of oxygen used to produce one ATP) than cold-acclimated fish (main effect of acclimation temperature; Table 1; Fig. 3c, Supplementary Material Fig. S7). There was also a tendency (at a one-tailed significance level; Table 1) for thyroid hormone and day length to influence the responses of P:O ratio to acute test temperature changes (three-way interaction), which may explain the significance of this interaction for ATP production scope (see below).

### Oxygen consumption scope did not predict ATP production

**Figure 4:**
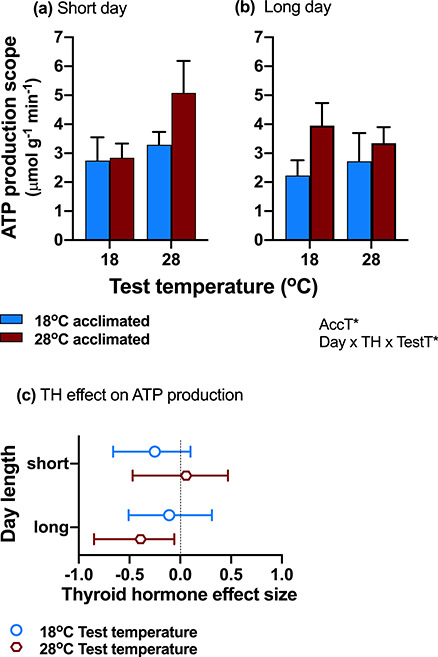
ATP production scope, calculated as the product between oxygen consumption scope and P:O ratio. ATP production scope was determined by a three-way interaction between hypothyroid treatment, day length [(**a**) short days, (**b**) long days] and acclimation temperature. Interestingly, ATP production scope showed the opposite patterns to MO_2_ scope, and warm-acclimated fish (red bars) had greater ATP production scope than cold-acclimated fish (blue bars) except at cold test temperatures on short days. Thyroid hormone reduced ATP production scope (negative effect sizes) when warm test temperatures (open red circles) were matched to long days, and (with relatively high confidence) when cold test temperatures (open blue circles) were matched to short days (**c**). The significant effects and interactions (thyroid status = TH, day length = day, acclimation temperature = AccT, and test temperature = TestT) are listed on the bottom right of each panel (^*^P < 0.05; ^**^P < 0.01: ^***^P < 0.0001). Means ± SE for normothyroid fish are shown in (**a**) and (**b**), and means ±95% CI are shown in (**c**). Sample size was *n* = 16–21 fish per treatment group

Unlike oxygen consumption scope, cold acclimation did not increase ATP production scope (i.e. estimated maximal-resting ATP-produced). Instead, warm acclimation significantly increased ATP production scope (main effect of acclimation; Table 1; Fig. 4a and b, Supplementary Material Fig. S9). Exposure to long days increased ATP production scope in warm-acclimated animals at both test temperatures, but following exposure to short days, warm acclimation increased scope only at the high test temperature (day length × acclimation temperature × thyroid status interaction; Table 1; Fig. 4a and b). Thyroid hormone decreased ATP production scope when day length and test temperatures matched, that is at cold test temperatures on short days and at warm test temperatures on long days (three-way interaction; Table 1; Fig. 4c).

### Impacts of warm temperature and short day length

**Figure 5:**
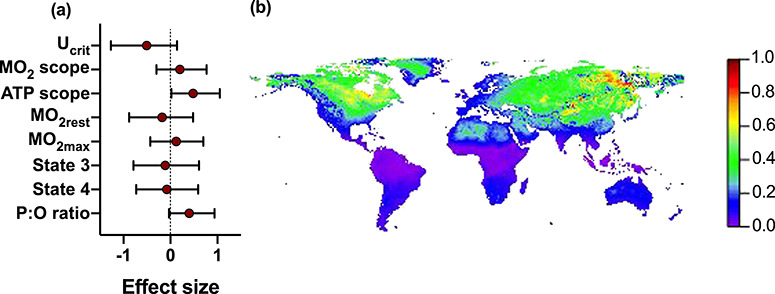
Effects of a temperature and day length mismatch. Climate warming may cause a mismatch between warm temperatures and short day length. The effect sizes (Cohen’s *d* ± 95% confidence intervals) of this mismatch (warm acclimation and test temperatures on a short day, compared to warm conditions on a long day) indicate that there is a high likelihood for *U*_crit_ to decrease, and for ATP production scope and P:O ratio to increase (**a**). The likelihood of occurrence of such a mismatch increases with increasing seasonality, and increasing future warming. We modelled these dynamics globally by multiplying current seasonality, estimated as the absolute temperature difference between July and January, by the projected climate warming under emission scenario RCP 8.5 for 2070 (**b**). The result was scaled to fall between 0 and 1, where 1 represents the highest impact. Note that the predictions do not include light at night.

The mismatch between warm temperatures (acclimation and test temperatures) and short day lengths is likely (effect size ±95% confidence intervals) to decrease swimming performance and increase mitochondrial efficiency (P:O ratios) and ATP production scope (Fig. 5a). We predicted that these effects will be greatest in areas with pronounced seasonality and high rates of predicted warming, which comprise most of North America and Asia (Fig. 5b); note, however, that we did not include light at night in the model predictions.

## Discussion

We have shown that exposure to mismatching seasonal light and temperature cues can alter whole-animal performance and energetics and that the effects of light and temperature were at least partly mediated by thyroid hormone. If the effects of light-temperature mismatches in warmer winters are more widespread than our study species, they are likely to be exacerbated by future climate change. Our predictions indicate that mid to high latitudes of the northern hemisphere, which experience substantial seasonality and future warming, may be particularly affected. Additionally, long days at cool temperatures, which can result from light at night—for example (Gaston, 2018), had a pronounced effect on thyroid hormone signalling, and can thereby influence locomotor performance and ATP production scope. Decreasing efficiencies of energy conversion from food-derived substrates to ATP in mitochondria decreased growth rates in brown trout (*Salmo salar*) (Salin *et al.*, 2019). If this relationship between ATP production efficiency and growth rate is widespread among animals, it can have pronounced negative ecological consequences that are mediated by light and temperature signals. However, increased ATP production efficiency can come at the cost of increased oxidative stress (Salin *et al.*, 2012), and these trade-offs have to be considered in interpreting our data. Similarly, the impact of light and temperature mismatches on locomotor performance can have ecological consequences. Locomotor performance is closely related to fitness, and even relatively small changes in sustained locomotion can be important ecologically (Dalziel and Schulte, 2012; Hillman *et al.*, 2014). The best-known effects of climate change on physiological functions are those mediated by temperature (Gunderson and Stillman, 2015). Here, we show that day length can modify thermal plasticity of locomotor capacity and ATP production, demonstrating that single environmental drivers do not act independently in modifying physiological rates, at least in mosquitofish. We note, however, that our experimental manipulation involved step changes in light regime representing seasonal extremes. This treatment can show that physiological responses are under photoperiodic control. Under natural situations, however, changes in light occur more gradually, which may modify physiological responses. Future work should address the potential implications of ramp changes in light cycles.

As hypothesized, short days enhanced swimming performance at the low temperature, and it is likely that these effects were mediated by thyroid hormone (Little and Seebacher, 2013). Thyroid hormone action promoted cold acclimation of sustained swimming in zebrafish exposed to short days, but it had the opposite effect on long days. This effect of thyroid hormone on swimming performance cannot be explained by thyroid-regulated changes in the ATP production machinery, because we showed with relatively high confidence that thyroid hormone decreased ATP production scope under these conditions. In addition to ATP availability, locomotor performance also relies on oxygen delivery by the cardiovascular system, and on skeletal muscle function (Dalziel and Schulte, 2012). We therefore suggest that, as previously described in zebrafish, thyroid hormone modifies swimming performance by regulating cold acclimation of cardiac capacity (Little and Seebacher, 2014) and muscle function (Little and Seebacher, 2013).

Climate change will lead to warmer winter and spring in many parts of the world, whereas the photoperiod (short days) remains unaltered. We showed that the positive effects of thyroid hormone on *G. holbrooki* are reduced under these circumstances, and locomotor performance decreases. This decrease in locomotor performance can have important repercussion on fitness, as locomotor capacity is important for various fitness-related behaviour, such as predator escape, and prey capture (Irschick and Garland, 2001; Irschick *et al.*, 2008). Global climate change also increases the frequency of extreme weather events, which may mismatch light and temperature over short periods. For example, cold spells in summer, as a result of heavy rainfalls for example, may disproportionally reduce swimming capacity compared to winter. Up to a point, however, fish may compensate behaviourally for these environmental changes by seeking thermal refugia, including cold-water refugia resulting from groundwater discharge (Wilbur *et al.*, 2020).

Interestingly, calibrating oxygen consumption with mitochondrial efficiency altered the conclusions from our treatments on whole-animal energy production. In particular, cold acclimation increased metabolic scope (oxygen consumption), but this was not paralleled by ATP production scope. Our oxygen consumption scope results suggest that cold-acclimated fish have more energy available for activity than warm-acclimated fish. A typical interpretation of this result would be that cold-acclimated fish are able to compensate for the negative thermodynamic effect of temperature by increasing their energy production. P:O ratios however revealed that cold-acclimated mitochondria are inefficient compared to warm-acclimated mitochondria. Once adjusted for P:O ratios, estimated ATP production scope shows that, on the contrary, cold-acclimated fish have less energy available for activity than warm-acclimated fish. Hence, drawing conclusions about energy availability from metabolic scope would lead to erroneous conclusions regarding the scope of energy production. Our results demonstrate that metabolic scope alone is not a reliable estimate of ATP production across environmental contexts and emphasize the importance of combining whole-animal and subcellular measurements of metabolism (Salin *et al.* 2015). We note, however, that our measurements are restricted to skeletal muscle, which does not necessarily represent other tissues. Nonetheless, skeletal muscle comprises up to 60% of total fish mass (Johnston *et al.*, 2011), so that even if its mitochondrial characteristics are different from those of other tissues, it will have a pronounced effect on whole-animal energetics.

The combined effect of light and temperature cues also modified mitochondrial bioenergetics. Rates of mitochondrial substrate oxidation (State 3) rates were higher under mismatched acclimation temperature and day length conditions, and on long days. Contrary to our hypothesis, therefore, the capacity for cold acclimation of substrate oxidation rates would be higher during summer than during winter. It may be that an increase in State 3 rates is not necessarily beneficial and comes at a cost, such as an increase in oxidative stress. Increased State 3 rates correlate positively with increased protonmotive force across the inner mitochondrial membrane, leading to higher rates of reactive oxygen species (ROS) production (Brand, 2000; Korshunov *et al.*, 1997). Excessive ROS production rates can disrupt cellular processes by causing oxidative damage to proteins, DNA and membranes (Costantini, 2019). Protonmotive force and ROS production can be decreased by increasing uncoupling of mitochondria via an increase in proton leakage (State 4 rate) (Brand, 2005). However, in our study, increases in State 3 rates were not accompanied by an increase in proton leakage, as State 4 rates were not affected by day length or temperature. ROS production also can be reduced by a decrease in State 3 rates, and the increase in State 3 rates in mismatched conditions therefore does not necessarily represent a regulated, beneficial response but could reflect a signal disruption. However, these speculations must be tested experimentally.

Increases in State 3 rates were not necessarily accompanied by an increase in mitochondrial efficiency, as day length had no significant effect on P:O ratios. In fish exposed to long days, the increase of State 3 rates in cold-acclimated fish was therefore not beneficial in terms of ATP production rates. This result demonstrates that increased State 3 rates should not be interpreted as increased performance. Our study showed that cold acclimation decreased mitochondrial efficiency. Several mechanisms could potentially explain the effect of thermal acclimation on P:O ratio independently from changes in State 3 and 4 rates (Brand, 2005). For example, high protonmotive force can cause a slippage in proton pumps (complex I, III and IV), in particular in cytochrome c oxidase (COX, complex IV), so that the H^+^/e^−^ stoichiometry is reduced, and less ATP is produced per electron transferred through the complexes of the respiratory chain (Kadenbach, 2003). The increase in State 3 rate induced by cold acclimation in fish exposed to long days could thereby also increase slip reactions in COX and reduce mitochondrial efficiency (P:O ratio).

Thyroid hormone increased resting oxygen consumption when acclimation and test temperatures matched. Thyroid hormones can influence metabolic rates in two ways. T3 can act genomically via thyroid receptors to increase catabolic enzyme concentrations in the longer term (days); this mechanism can potentially increase both maximal oxygen consumption and ATP production rates, depending on underlying mitochondrial efficiencies (Kadenbach, 2003). The second and short-term mechanisms involves binding of T2 to COX, thereby preventing allosteric inhibition of COX by ATP, which would lead to increased membrane potential and basal metabolic rates (in mammals), but decreased efficiency of mitochondria (i.e. decreased P:O ratio) (Kadenbach, 2003; Lanni *et al.*, 2016). Our data indicate that these dynamics are modulated by the interaction between temperature and light. For example, thyroid hormone was more likely to decrease ATP production scope when test temperature and day length matched, indicating that, under these conditions, T2 interacted with COX to reduce efficiency. These mitochondrial dynamics are important ecologically, because they can determine ATP production and therefore growth and other ATP-consuming processes (Salin *et al.*, 2019). However, the potential increase in oxidative stress can also cause damage to DNA, proteins and membranes, which can lead to disease and cell death (Costantini, 2019; Zuo *et al.*, 2015).

Mismatches between temperature and light occur at a global scale, and mid to high latitudes in Asia and North America are likely to be particularly affected. It is likely that the physiological responses to this mismatch are similar in other species as well, given the high evolutionary conservatism and similarity in function of mitochondria and skeletal muscle at least among vertebrates. Anthropogenic environmental changes in temperature and light regimes may therefore influence the fundamental processes underlying energetics and movement via their cellular effect on mitochondria and hormone signalling. We suggest that future research should focus on disentangling how temperature and light modify the complex dynamics of mitochondrial and muscle function and determine their consequences for reproduction, growth and dispersal.

## Supplementary material

Supplementary material is available at *Conservation Physiology* online.

## Funding

This work was supported by Australian Research Council Discovery Grant DP180103036 to F.S. A.L.R. was supported by a University of Sydney International Scholarship.

## Supplementary Material

CONPHYS-2019-153_Supporting_data_revision
