# Supplementary material for: Mismatched light and temperature cues disrupt locomotion and energetics via thyroid-dependent mechanisms
Source: Conserv Physiol. 2020 Jun 11;8(1):coaa051. doi: 10.1093/conphys/coaa051 (PMC7287392; doi:10.1093/conphys/coaa051)
Supplement: CONPHYS-2019-153_Supporting_data_revision [file conphys-2019-153_supporting_data_revision.docx]

**Supporting data**

**Mismatched light and temperature cues disrupt locomotion and energetics via thyroid-dependent mechanisms**

**Amélie Le Roy, Frank Seebacher***

*School of Life and Environmental Sciences A08, University of Sydney, NSW 2006, Australia*

*author for correspondence:

email: frank.seebacher@sydney.edu.au

phone: +61 2 93512779

**Supplementary Figures**

**Figure S1** Verification of the efficacy of the hypothyroid treatment. U_crit_ in cold acclimated fish exposed to short day are shown separately for control fish (black bars), hypothyroid fish (grey bars) and hypothyroid fish that were daily supplemented with 10 nmol.l-1 T2 and T3 (clear bars). Thyroid status (control, hypothyroid, supplemented with T2 and T3) had a significant effect (p < 0.0001), and U_crit_ was significantly lower in the hypothyroid group than the control (p = 0.01) or supplemented groups (p < 0.0001). However, there was no difference between the latter two groups (p = 0.088), confirming the efficacy of our hypothyroid treatment. Sample size was n = 11-16 per treatment group.

**Figure S2** U_crit_ in control and hypothyroid fish. U_crit_ was modified by the interaction between day length and test temperature, by the interaction between acclimation temperature and test temperature, and by the three-way interaction between day length, acclimation temperature and thyroid status. Results are shown as means ± s.e., and separately for fish exposed to short days (a, c) or long days (b, d), and for cold- (a, b) and warm-acclimated (c, d) fish. Each panel shows results for control fish (black bars) and hypothyroid fish (grey bars). Sample size was n = 16-21 fish per treatment group.

**Figure S3** Resting oxygen consumption rates in control and hypothyroid fish. The three-way interaction between test temperature, acclimation temperature, and thyroid status had a significant effects on oxygen consumption (MO_2_). Results are shown as means ± s.e.m. and separately for fish exposed to short days (a, c) or long days (b, d) and for cold- (a, b) and warm (c, d) acclimated fish. Each panel shows results for control fish (black bars) and hypothyroid fish (grey bars). Sample size was n = 16-21 fish per treatment group.

**Figure S4** Maximal oxygen consumption rates in control and hypothyroid fish. The main effects of test temperature and acclimation temperature had significant effects on oxygen consumption. (MO_2_). Results are shown as means ± s.e.m. and separately for fish exposed to short days (a, c) or long days (b, d) and for cold- (a, b) and warm (c, d) acclimated fish. Each panel shows results for control fish (black bars) and hypothyroid fish (grey bars). Sample size was n = 16-21 fish per treatment group.

**Figure S5** Oxygen consumption scope in control and hypothyroid fish. Test temperature and acclimation temperature had a significant main effect on MO_2_ scope. Results are shown as means ± s.e.m. and separately for fish exposed to short days (a, c) or long days (b, d) and for cold- (a, b) and warm (c, d) acclimated fish. Each panel shows results for control fish (black bars) and hypothyroid fish (grey bars). Sample size was n = 16-21 fish per treatment group.

**Figure S6** State 3 mitochondrial respiration rates. The interactions between day length and acclimation temperature, and between day length and test temperature significantly affected state 3 rates. Results are shown as means ± s.e.m. and separately for fish exposed to short days (a, c) or long days (b, d) and for cold- (a, b) and warm (c, d) acclimated fish. Each panel shows results for control fish (black bars) and hypothyroid fish (grey bars). Sample size was n = 16-21 fish per treatment group.

**Figure S7** State 4 mitochondrial respiration rates. There were no significant effects of any experimental factors on state 4 rates. Results are shown as means ± s.e.m. and separately for fish exposed to short days (a, c) or long days (b, d) and for cold- (a, b) and warm (c, d) acclimated fish. Each panel shows results for control fish (black bars) and hypothyroid fish (grey bars). Sample size was n = 16-21 fish per treatment group.

**Figure S8** P:O ratio in control and hypothyroid fish. Acclimation temperature had a significant effect on P:O ratios. Results are shown as means ± s.e.m. and separately for fish exposed to short days (a, c) or long days (b, d) and for cold- (a, b) and warm (c, d) acclimated fish. Each panel shows results for control fish (black bars) and hypothyroid fish (grey bars). Sample size was n = 16-21 fish per treatment group.

**Figure S9** ATP production scope in control and hypothyroid fish. Acclimation temperature, and the three-way interaction between day length, test temperature, and thyroid status had significant effects on ATP production scope. Results are shown as means ± s.e.m. and separately for fish exposed to short days (a, c) or long days (b, d) and for cold- (a, b) and warm (c, d) acclimated fish. Each panel shows results for control fish (black bars) and hypothyroid fish (grey bars). Sample size was n = 16-21 fish per treatment group.
